# The first mitochondrial genome of *Tetraclita kuroshioensis* (Crustacea: Sessilia) from China: insight into the phylogeny within Cirripedia

**DOI:** 10.1080/23802359.2021.1891984

**Published:** 2021-03-18

**Authors:** Nanjing Ji, Tian Ge, Sheng Mao, Mengjuan Zhang, Ning Mao, Yuefeng Cai, Xin Shen

**Affiliations:** a Jiangsu Institute of Marine Resources, Jiangsu Key Laboratory of Marine Biotechnology, Jiangsu Ocean University, Lianyungang, PR China; b Co-Innovation Center of Jiangsu Marine Bio-industry Technology, Jiangsu Ocean University, Lianyungang, PR China

**Keywords:** *Tetraclita kuroshioensis*, Tetraclitidae, Phylogenetic, Cirripedia

## Abstract

We determined the first mitochondrial genome of *Tetraclita kuroshioensis* from China. The mitochondrial genome of *T. kuroshioensis* was found to be 15,175 bp in length and consisted of 13 protein-coding genes (PCGs), 22 tRNAs, and 2 rRNAs. The longest non-coding region was 425 bp in length. Phylogenetic analysis showed that *T. kuroshioensis* clustered with *T. serrata* and then clustered with *T. squamosa squamosa* with high bootstrap value (BP = 100). In the future, sequencing of additional mitochondrial genomes should provide additional insights into the deep phylogeny of Cirripedia.

Cirripedia (Crustacea) are important model organisms in marine ecology and biofouling studies (Cao et al. 2013). *Tetraclita kuroshioensis* samples were collected from Haikou, China (20.06 N, 110.35 E) on 10 November 10 2019 and were deposited at the Museum of Jiangsu Ocean University (https://www.jou.edu.cn/, voucher number: TkuHK-001). Genomic DNA extraction was performed using the TIANamp Marine Animals DNA Kit according to the manufacturer’s instructions (TIANGEN, Beijing, China). The mitochondrial genome of *T*. *kuroshioensis* was sequenced and annotated according to a previous study (Chen et al. 2019). Briefly, the DNA fragments were obtained by PCR amplification and assembled using SeqMan software in the DNAStar package (DNASTAR Inc., Madison, WI). Gene annotation was conducted by MITOS (Bernt et al. 2013) and tRNAscan-SE (Chan and Lowe 2019).

The mitochondrial genome of *T. kuroshioensis* is a typical circular DNA molecule (15,175 bp), encoding 13 protein-coding genes (PCGs), 22 tRNAs, and 2 rRNAs (GenBank accession number: MW298526) (Supplementary Table S1). Overall, the A + T content was 66.7% which is in the typical range of other metazoan mitochondrial genomes (Saccone et al. 1999). Thirteen genes are encoded on the light strand, while the other genes are located on the heavy strand. The cumulative length of all non-coding regions was found to be 615 bp and the longest one was located between *srRNA* and *trnK* (425 bp), which was assumed to be the control region. In comparison with the pancrustacean genome organization (von Reumont et al. 2012), seven conserved blocks were found in the mitochondrial genome of *T. kuroshioensis* (*cox1- trnL2-cox2*, *trnD-atp8-atp6-cox3-trnG-nad3*, *trnR-trnN*, *trnF-nad5-trnH-nad4-nad4L*, na*d6-cob-trnS2*, *nad1-lrRNA-trnV-srRNA*, and *trnM-nad2-trnW*), which was consistent with our previous studies (Ge et al. 2019).

In this study, phylogenetic analysis was conducted based on the nucleotide sequences of 13 PCGs using a maximum-likelihood method in MEGA version 7.0.25 (Kumar et al. 2016). The phylogenetic tree showed that *T. kuroshioensis* clustered with *T*. *serrata* and then clustered with *T*. *squamosa squamosa* into a branch with high bootstrap value (BP = 100), and Tetraclitidae was not monophyletic. In addition, we also found Pyrgomatidae, Balanidae, and Archaeobalanidae to be paraphyletic clades which were consistent with previous studies (Song et al. 2017; Tsang et al. 2017; Chen et al. 2019; Feng et al. 2020) (Figure 1). The sequencing of more mitochondrial genomes data shall further our understanding on the phylogeny within Cirripedia.

**Figure 1.**
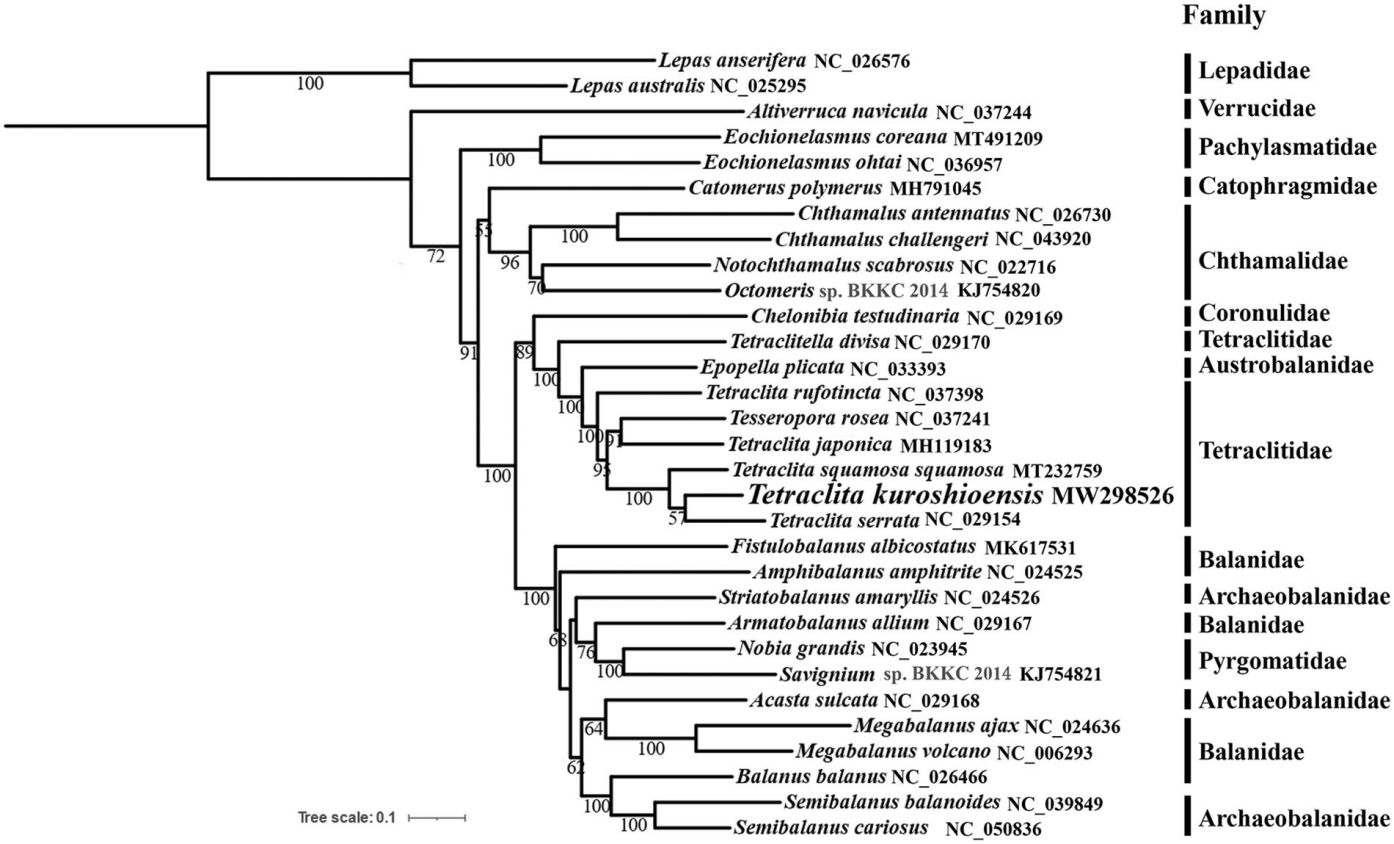
The maximum-likelihood phylogenetic tree based on 13 PCGs nucleotide sequences of *Tetraclita kuroshioensis* and other mitochondrial genomes from Cirripedia.

## Data Availability

The genome sequence data that support the findings of this study are openly available in GenBank of NCBI at (https://www.ncbi.nlm.nih.gov/) under the accession no. MW298526. The associated BioProject, SRA, and Bio-Sample numbers are PRJNA692667, SRP302080, and SAMN17360264, respectively.
